# Anti-PAI-1 Monoclonal Antibody Inhibits the Metastasis and Growth of Esophageal Squamous Cell Carcinoma

**DOI:** 10.7150/jca.77888

**Published:** 2023-01-01

**Authors:** Yutian Zheng, Lin Meng, Like Qu, Chuanke Zhao, Lixin Wang, Caiyun Liu, Chengchao Shou

**Affiliations:** Key Laboratory of Carcinogenesis and Translational Research (Ministry of Education/Beijing), Department of Biochemistry and Molecular Biology, Peking University Cancer Hospital & Institute, Beijing 100142, China.

**Keywords:** Esophageal squamous cell carcinoma (ESCC), Plasminogen activator inhibitor (PAI-1), Monoclonal antibodies (mAb), Anti-tumor growth, Anti-metastasis

## Abstract

Plasminogen activator inhibitor (PAI-1) is highly expressed in esophageal squamous cell carcinoma (ESCC) and strongly contributes to metastasis, making it a potential target for ESCC therapy. However, the antibodies and inhibitors targeting PAI-1 have not shown good therapeutic effect in the *in vivo* experiments yet. Here, we generated a panel of novel monoclonal antibodies (mAbs) against PAI-1. Analysis of PAI-1 expression in 90 tissue specimens and 128 serum specimens from ESCC patients with these mAbs confirmed that PAI-1 levels was significantly correlated with metastasis and poor survival. In addition, we found that high PAI-1 expression contributed to the enhanced motility and invasiveness of two ESCC cell lines. Next, mAb-1E2 and mAb-2E3, which have highest affinity with PAI-1, were shown to possess strong inhibitory effects on ESCC migration and invasion. Anti-tumor and anti-metastatic effects of mAb-2E3 were further demonstrated in the experimental animal models. Finally, LRP1 was identified as key factor mediating the pro-invasive function of PAI-1 and the anti-invasive capacity of mAb-2E3 in ESCC cells. The mAb-2E3 markedly decreased STAT1 phosphorylation levels and blocked the binding between PAI-1 and LRP1-ClusterII domain. Collectively, mAb-2E3 developed by our lab may be an effective antibody drug which can be used for anti-metastatic therapy in ESCC.

## Introduction

Esophageal squamous cell carcinoma (ESCC) is one of the most common types of cancer in Chinese cancer patients. It is difficult to be diagnosed at early stage and easy to invade and metastasize, resulting in poor prognosis and high mortality 1. Therefore, reducing the incidence of metastasis is a key research direction in the treatment of ESCC. However, the therapeutic options for the recurrent and metastatic ESCC are limited. There were only 40-50% remission rates have been reported for therapy with Pembrolizumab or Camrelizumab, which failed to meet the clinical needs 2,3. At present, the anti-angiogenic tyrosine kinase inhibitor Antironib, which is more effective in inhibiting ESCC metastasis, has entered phase III clinical trials 4. In addition, the response rate of Nimotuzumab combined with Paclitaxel reached 51.8%, and a randomized phase III clinical study are ongoing 5. Combination immunotherapy with antibodies against PD-1 or PD-L1 has also been developed 6,7,8. However, many patients with metastatic ESCC receive only one line of treatment, and resistance may be inevitable, meaning that they do not have the opportunity to benefit from novel antibodies 9. Therefore, it's critical to change the treatment landscape of recurrent and metastatic ESCC to search for important biomarkers and develop specific targeted antibodies 6,7,8,9,10.

The fibrinolytic system plays an important role in tumor metastasis. The role of the uPA/uPAR system in promoting tumor metastasis has been demonstrated since uPA was the first identified protease involved in tumor-associated fibrinolysis 11,12,13. As the principal inhibitor of uPA activity, it would logically follow that PAI-1 would decrease tumor invasiveness. PAI-1 is a single-chain exocrine glycoprotein containing 379 amino acid residues, which belongs to the serine protease inhibitor superfamily 14. Through its unique RCL structure (Reactive center loop), PAI-1 irreversibly binds to the double-stranded uPA covalently in a ratio of 1:1, thereby inhibiting uPA activity and reducing ECM remodeling 15,16,17. Contrary to these *in vitro* findings, PAI-1 was not a potent inhibitor of tumor metastasis. The high levels of PAI-1 can exert the opposite effect by interacting with an important component of the ECM, vitronectin, competing with uPAR and integrins to bind to the central adhesion site, thus inducing cell shedding and migration 13,18. In recent years, PAI-1 has been found to be highly expressed in a variety of solid tumors, especially in gastric adenocarcinoma, esophageal cancer, colorectal cancer and plays an important role in metastasis. Several lines of evidence support that high expression of PAI-1 is associated with metastasis of ESCC and poor prognosis 19,20,21,22. Cancer-associated fibroblast-derived PAI-1 promotes cancer cell invasion and macrophage migration 23, while overexpression of PAI-1 also promotes cell proliferation, migration and invasion in ESCC 24.

PAI-1 binds to a variety of protein, such as PA, VTN and LRP1 by changing conformation states, and most of the PAI-1 complex could promote tumor metastasis 11,13,18. Vitronectin binds to PAI-1 through the helices hD, hE, and hF in the flexible joint region. LRP1 endocytoses PAI-1 or PAI-1-/uPA/tPA mainly through recognition with several key residues of helix hD, such as K60, K69, R76 and R138 25,26. In response to this feature, small molecule inhibitors and antibodies targeting PAI-1 have been developed. The most reported is PAI-039, which inhibits proliferation, angiogenesis and accelerates apoptosis in various tumor cells. IMD-4482 exhibits good anti-invasive ability in ovarian cancer 26,27,28. The natural product oxymatrine could inhibit migration and proliferation of colorectal cancer cells through inhibition of PAI-1 and the TGF-β1/Smad signaling pathway 29,30. Nonetheless, these remain impractical for cancer treatment due to their short *in vivo* half-lives. In addition, most inhibitors have only limited *in vitro* activity, therefore it is difficult to enter clinical trials 31,32.

In this study, we generated a panel of PAI-1-specific mAbs. With these novel mAbs, increased PAI-1 levels in serum and tissue samples were found in ESCC, which were closed associated with lymph node metastasis. We further evaluated anti-metastatic effect of mAb-2E3 in the *in vitro* and *in vivo* assays. Mechanistically, we found that mAb-2E3 was able to bind the active form of PAI-1, inhibit metastasis of esophageal cancer in an LRP1-dependent manner, and block the binding between LRP1-Cluster II and PAI-1.

## Materials and Methods

### Cells and cell culture

The human ESCC cell lines KYSE-140, KYSE-410, KYSE-510 were obtained from ATCC. YES-2, TE12, KYSE30lm3, KYSE30luc, KYSE450lm2, and KYSE450luc were gifted by Prof. Zhihua Liu (Cancer Institute and Cancer Hospital, Chinese Academy of Medical Sciences and Peking Union Medical Collage). The KYSE30luc and KYSE450luc cell lines were obtained by labeling the KYSE30 and KYSE450 cells with luciferase reporter genes, which were injected into immunodeficient mice (SCID/Beige) via the tail vein. After 2-3 months, the lungs were taken out under aseptic conditions, minced and digested, and the primary culture of ESCC cells in lung metastases was carried out. These cells were injected secondly into SCID/Beige via the tail vein. KYSE30luc cells were screened for three rounds, while KYSE450luc cells were screened for two rounds, and finally a subset of cells with strong lung metastatic ability (KYSE30lm3 and KYSE450lm2) were obtained and cultured in RPMI-1640 medium supplemented with 15% FBS and 100 μg/mL G418 33. KYSE-140, KYSE-410, KYSE-510, YES-2, and TE12 were maintained in RPMI-1640 supplemented with 10% FBS. SP2/0 myeloma cells were maintained in RPMI-1640 supplemented with 20% FBS. 293T cells were maintained in DMEM supplemented with 10% FBS. All cells were grown at 37°C with 5% CO2, 95% air atmosphere.

### Small interfering RNA (siRNA) synthesis, vector construction, and transfection

The full-length sequence of human PAI-1 eukaryotic plasmid (pCDNA3.0-myc-SERPINE1) and prokaryotic plasmid (pGEX-4T1-GST-SERPINE1) were synthesized by Sangon Biotech (Suzhou, China). The vector pCDNA3.0-myc and pGEX-4T1-GST were purchased from Public Protein/Plasmid Library (Nanjing, China). The siRNAs, including si-PAI-1#1 (GCACCACAGACGCGAUCUUTT), siPAI-1-#2 (CCAUGAUGGCUCAGACCAATT) and si-LRP1#1 (GCCGAUCGAUCUUCACAAATT), siLRP1#2 (GGUCCAACUACACGUUACUTT), siLRP1#3 (GCGCAUCGAUCUUCACAAATT) and control siRNA (UUCUCCGAACGUGUCACGUTT) were synthesized by GenePharma (Shanghai, China).

Plasmids were transfected with Lipofectamine2000 (Invitrogen, American) for KYSE30luc and KYSE450luc cells. siRNA was transfected with buffer Mate (GenePharma, Shanghai, China) for tumor cells. All transfections were performed according to the manufacturer's instructions. The cells were collected and used for Western blot assays 48h after transfection.

### Antibodies and proteins

Antibodies against PAI-1 (ab222754, 1:500; ab125687, 1:100), anti-VTN (ab45139, 1:2000), anti-LRP1 (ab168454, WB 1:1000, IP 1:100), anti-SMAD2 (phosphor S467) (ab280888), anti-SMAD2(ab40855), anti-SMAD3 (phosphor S423+S425) (ab172202), anti-SMAD3 (AB40854) were purchased from Abcam. Anti-PLAT (sc-515562, 1:50) and anti-PLAU (sc-59727, 1:500) were purchased from Santa cruz. Anti- Phospho-STAT1 (Tyr701) (7649T), anti-STAT1 (14994T), anti-Phospho-p44/42 MAPK (Erk1/2) (Thr202/tyr204) (4370), anti p44/42 MAPK (Erk1/2) (4695), anti-Phospho-Akt (Ser473) (4060), anti-Akt (14702) anti-phospho-PI3 Kinase p85 (Tyr458)/p55 (Tyr199) (4228), anti-PI3 Kinase p85(4257), anti-E-Cadherin (3195T), anti-N-Cadherin (13116T), anti-phospho- NF-κB p65 (Ser536) (3033s), anti- NF-κB p65 (8242) were purchased from CST. Anti-Anti-GAPDH (10494-1-AP) was purchased from Proteintech. His tagged human PAI-1 (10296-H08H) was purchased from Sino Biological. His-PLAU (PLU-H5229) was purchased from Acro. Human Lys-plasminogen (HPG2002), Glu-plasminogen (HPG2001) and S-2251 (100-01) were purchased from Enzyme Research Laboratories. Z-GGR-AMC was purchased from A+ peptide (shanghai). Recombinant Human LRP1-Cluster II-Fc Chimera protein (2368-L2) was purchased from Bio-Techne.

### RNA extraction and quantitative RT-PCR (qPCR)

Cells were harvested in Trizol® reagent (Invitrogen), and total RNA was isolated according to the manufacturer's instructions. Single-stranded cDNA was synthesized from 5 µg total RNA using M-MLV reverse transcriptase (Invitrogen), with an oligo(dT)18-mer and Random primers, in a final reaction volume of 20 μL. The resulting complementary DNA was subjected to Real-time PCR using SYBR Green qPCR Master Mix (Promega). GAPDH was used as an internal standard. Primers used for PAI-1, forward: 5'-TGGCTCAGACCAACAAGTTCAA-3', reverse: 5'-GGCAGTTCCAGGATGTCGTAG-3'; for LRP1, forward: 5'-CTATCGACGCCCCTAAGACTT-3', reverse: 5'- CATCGCTGGGCCTTACTCT-3'; for STAT1, forward: 5'- CAGCTTGACTCAAAATTCCTGGA-3'; reverse: 5'- TGAAGATTACGCTTGCT TTTCCT -3'; for GAPDH, forward: 5'-CATCAAGAAGGTGGTGAAGCAG-3', reverse: 5'-CGTCAAAGGTGGAGGAGTGG-3'.

### Migration and Invasion assay

For migration assay, 5×10^4^ KYSE30lm3/KYSE30luc and 2×10^5^ KYSE450lm2/KYSE450luc cells were plated in 24-well Transwell plates with inserts (8-μm pore size, Corning) and were incubated at 37°C for 24h. Invasion assays were carried out in a 24-well Transwell unit on polycarbonate filter coated with Matrigel (Corning). Cell inserts were fixed with 4% PFA for 30 min, followed by PBS wash and 1% crystal violet staining to allow visualization. Nine random fields were captured per sample at 10× magnification. The ratio of the average stained area to the field of view of each sample was calculated by Image J. All experiments were performed in triplicate.

### Wound-healing assay

The cells were seeded into a 6-well plate and allowed to grow to 90% confluence. Cell were cultured in serum-free RPMI-1640 media and scratched using sterile 10-μL pipette tips and washed for three times with PBS to remove cell debris. Cells migrated into wound surface and the average distance of migrating cells were determined under an inverted microscopy after 12 and 24 hours. All experiments were performed in triplicate.

### Cell proliferation (CCK8) assay

Cells in logarithmic growth phase were seeded into a 96-well plate at a density of 5×10^3^ cells/well. The viability of cells was determined using Cell Counting Kit-8 systems (Dojindo Laboratories, Japan). The experiments were performed in triplicate.

### Generation and purification of anti-PAI-1 monoclonal antibodies

BALB/C mouse were used for immunization. PAI-1 plasmid mixed with *In vivo*-jetPEI (Polyplus) transfection reagent was used as antigen. Ten days after the last boost the sera of the immunized mice were tested for the PAI-1 specific antibody by ELISA, and the spleen cells were isolated and fused with myeloma cells to obtain hybridomas. Limited dilution was performed as standard step to selected hybridoma cell clones. We used GST-PAI-1 prokaryotic protein and GST protein (2 μg/mL) as coating antigen of primary screening, and His-PAI-1 (400 ng/mL) recombinant protein as coating antigen in the next few rounds of screening. Antibodies were prepared by intra-peritoneal injection of hybridomas into 8-week-old BALB/C female mice. One week before the hybridoma injection, 500 μL of incomplete Freund's adjuvant was injected. After 10-15 days, the ascites was collected, and protein G agarose beads were used for antibody purification. After being concentrated by PEG20000, antibodies were dialyzed by PBS and identified with SDS-PAGE.

### Immunoprecipitation and Co-immunoprecipitation

KYSE30lm3 cells were lysed with IP buffer (50 mM Tris-HCl, pH 7.5, 150 mM NaCl ,1mM EDTA and 0.5% NP-40) containing protease inhibitors (Roche, USA), and the lysates were used for IP with appropriate antibodies as well as protein G-Sepharose (GE Healthcare, USA). The precipitants were separated by SDS-PAGE and WB with specific antibodies conjugated to horseradish peroxidase (Biodragon, China). Visualization was achieved with chemiluminescence.

### IHC staining

Tissue microarrays containing 90 ESCC and normal esophageal tissues were purchased from Shanghai outdo biotech. The slides were incubated with 3% H_2_O_2_ for 10 min. After antigen retrieval with pH 9.0 EDTA, the slides were blocked with 5% goat serum (ZLI-9056, ZSGB-BIO) for 1 h. For human tissue specimen, the slides were incubated with mAb-PAI-1-15C2 or antibody of PAI-1 (ab125687) as primary antibodies and then incubated with HRP-labeled goat anti-mouse/rabbit IgG. The intensity of PAI-1 expression was graded as “-”, “+” weak cytoplasmic staining; “±” strong staining in <30% of cancer cells, “++” strong staining in more than 30% of cancer cells. 10% or more of the stained area positive is considered positive for each specimen. The level of PAI-1 was judged according to the shade of brown.

### Western blot assay

For western blot analysis, cells were collected and lysed with 40 mM Tris-HCl (pH 7.4), 150 mM NaCl, 1% (v/v) Triton X-100, 1 x cocktail of protease inhibitors and PMSF. 20 μg proteins were separated and transferred to a NC membrane. After blocking with 5% non-fat milk, the membranes were incubated with primary antibody at 4°C overnight. HRP-conjugated sheep anti-rabbit or anti-mouse IgG secondary antibodies (Vector, Burlingame, CA) were incubated for 1h at room temperature. The protein bands were detected using the Super Enhanced chemiluminescence detection kit (Applygen Technologies Inc, Beijing, China).

### Sandwich ELISA

Anti-PAI-1 mAbs were purified on protein-G and a functional pair of anti-PAI-1 mAbs was identified for the development of a sandwich ELISA. In brief, the capture mAb (1E2, IgG2b) was immobilized at 2 μg/mL on black 96-well high-binding polystyrene plates at 5 μg/mL in 0.1 M carbonate buffer (pH 9.4). Plates were washed repeatedly in Tris-buffered saline with 0.1%Tween-20 (TBST, pH 7.2) and blocked with 10% non-fat milk. The ESCC serum samples (obtained from the Tissue Bank of Peking University Cancer Hospital) were diluted in TBST containing 0.5% BSA and added to wells for 2 h. The detection mAb (2E3, IgG2b) conjugated to HRP was added at 1 μg/mL for 1 h. TMB was added and OD450 nm was recorded. All reactions were performed at RT with a minimum of three replicates.

### PAI-1 activity assay and uPA neutralization assay

For Z-GGR-AMC assay, 25 μL His-PAI-1 protein was incubated with 25 μL, 2 μg/mL uPA (0.05 M Tris·HCl, 0.01M NaCl pH 7.4) at 37 °C 30 min, 50 μL Z-GGR-AMC (100 μM) was added and the PAI-1 activity was measured by recording the excitation wavelength 380nm.

For plasminogen assay, 50 μL His-PAI-1 protein (200 nM) was incubated with 50μl of uPA (5 IU/mL) at 37℃ for 15 min in wells of a microtiter plate. 100μl of a solution containing human Glu-plasminogen (1 mM) and S-2251 (0.6 mM) was added. The PAI-1 activity was measured by recording the absorbance change at 405 nm. For antibody neutralization experiments, 25 μL His-PAI-1 protein (200 nM) was incubated with 25 μL antibodies from 0 μg/mL to 20 μg/mL at RT 2h, then 25 μL mixture was added in 25 μL uPA (5 IU/mL) at 37°C 30 min, 100 μL of a solution containing human Glu-plasminogen (1 mM) and S-2251 (0.6 mM) was added and the residual active PAI-1 was measured by recording the absorbance change at 405 nm after 37°C 30min.

### MAb-2E3 blocking the binding of LRP1-Cluster II-Fc and PAI-1

For PAI-1 and LRP1-Cluster II-Fc binding experiments, ELISA plates were coated with LRP1-Cluster II-Fc protein. 100 μl/well of LRP1-Cluster II-Fc protein diluted to 100 ng/mL with PBS was added to the plate and incubated at 37°C for 2 h. After discarding the coating solution, 300 μl/well of 3% BSA was used for blocking for 2 h at room temperature. After the plates were washed 5 times with 0.05% PBST, 100 μl of PAI-1 protein diluted in PBS was added according to the concentration gradient, and two replicate wells were set up at each concentration. After 1 h of binding at room temperature, the PAI-1 binding solution was discarded, the plates were washed 5 times with 0.05% PBST, then 100 μl of anti-His-HRP antibody was added and incubated at room temperature for 1 h. After discarding the antibody, plates were washed 5 times with 0.05% PBST. 100 μl per well of TMB was added and color was developed at room temperature over light until the positive control reaches a certain color. The reaction was terminated by 50 μl of 12.5% H_2_SO_4_, and OD450 reading was performed. EC50 (Concentration for 50% of maximal effect) values were obtained by Graph prism statistics and a Dose-response-Stimulation variable slope (four parameters) model was constructed.

For the experiment of antibody blocking, 200 ng/mL PAI-1 and an equal volume of mAb-2E3 were incubated together at room temperature for 1 h. Then the mixture of them was added to the plate coated with LRP1-Cluster II-Fc protein, and incubated at room temperature for 1 h. The subsequent steps were the same as the PAI-1 and LRP1-Cluster II-Fc protein binding experiment. IC50 (half maximal inhibitory concentration) values were obtained by Graph prism statistics and a Dose-response-inhibition variable slope (four parameters) model was generated.

### *In vivo* assay

Six-week old male SCID/Beige mice were purchased from Charles River (Beijing, China). Animal experiments were approved by the Biomedical Ethical Committee of Peking University Cancer Hospital & Institute and performed along institutional animal welfare protocols concordant with the NIH guidelines. For tumor metastasis model, mice were randomized into two groups, KYSE30lm3 cells (2×10^6^) were injected into the tail vein of mice, which were subsequently killed 8 weeks later. For tumor growth model, KYSE30lm3 cells (2×10^6^) were injected subcutaneously into the flanks of mice, which were killed 4 weeks later. All mice were treated with mAb-2E3 (10 mg/kg, 40 mg/kg) or mIgG (40 mg/kg) twice a week.

### Statically analysis

The SPSS (version 26) software package (SPSS Inc, Chicago, IL) was used for statistical analysis. The data are expressed as the mean ± SD, and the data from specific experiments were compared by one-way ANOVA or Student's t-test or χ^2^ test. GraphPad Prism 6 software (GraphPad Software, San Diego, CA, USA) was used for statistical figure or chart display. Chi-square analysis was used for differential expression of PAI-1 in ESCC tissue microarrays. All experiments were repeated at least three with consistent results. *P*-value <0.05 was considered statistically significant.

## Results

### Generation of PAI-1 mAbs and characterization of their specificities

**Figure 1:**
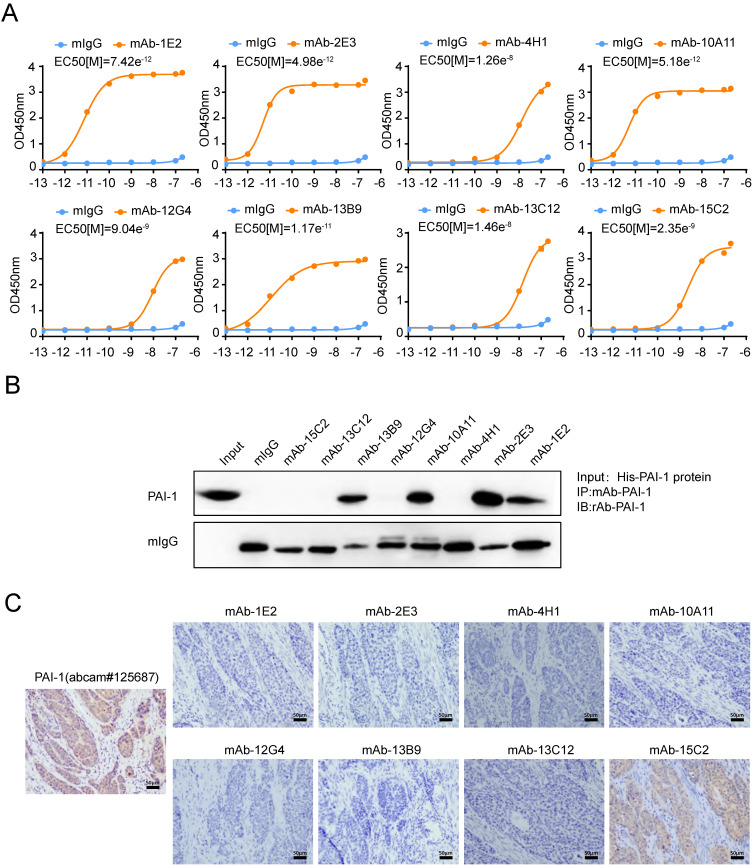
** Screening of human specific anti-PAI-1mAbs. A.** The binding affinity of anti-PAI-1 mAbs to His-PAI-1 proteins was measured by ELISA. PAI-1 protein was coated in the 96-well plate at 50 μl/well overnight. Anti-PAI-1 mAb were added to the wells along gradient of concentration 10-fold increments ranging from e^-13^M to e^-7^ M. **B.** Immunoprecipitation of eukaryotic PAI-1 proteins with anti-PAI-1 mAbs and control mIgG. **C.** Immunohistochemical staining of ESCC tissue sections with anti-PAI-1 mAbs.

Since PAI-1 was expected to be a potential anti-metastasis target for ESCC, we then sought to generate antibodies against PAI-1. Mixture containing PAI-1 eukaryotic plasmid and PEI transfection reagent were used to immunize mice. After hybridoma fusion and clone screening, we obtained eight specific antibodies which could react with GST-PAI-1 protein (Supplement Fig. S1A). The half maximal effective concentration (EC50) of the antibody was calculated by doubling the dilution and that of mAb-2E3 is the lowest (4.983e^-12^) (Fig. 1A), suggesting the strongest binding ability to PAI-1. In addition, the EC50 values ​​of mAb-1E2 and 10A11 also reached the order of e^-12^. We further examined the recognition and binding specificity of the mAb to PAI-1 from eukaryotic (Fig. 1B, Supplement Fig. S1B) and prokaryotic sources (Supplement Fig. S1C-D). The antibodies which can specifically bind to PAI-1 or recombinant protein in the cell lysate include mAb-1E2, mAb-2E3, mAb-10A11 and mAb-13B9, and mAb-2E3 has the strongest binding ability. By using the plasminogen system and the uPA substrate Z-GGR-AMC system, we confirm the activity of the PAI-1 eukaryotic protein (Supplement Fig. S2A-B). Hence, these anti-PAI-1 mAbs could recognize active form of PAI-1 protein. Results of immunohistochemical staining showed that mAb-15C2 had advantages over other antibodies in recognizing PAI-1 protein in ESCC tissues (Fig. 1C). Next, we carried out HRP enzyme labeling and pairing of high-affinity antibodies to develop the double-monoclonal antibody sandwich ELISA system for PAI-1 detection. HRP-mAb-2E3 and mAb-1E2 was respectively screened as the detection antibody and the capture antibody (Supplemental Fig. S3A-B).

### PAI-1 overexpression in ESCC tissues and serum correlates with ESCC aggressiveness

**Figure 2:**
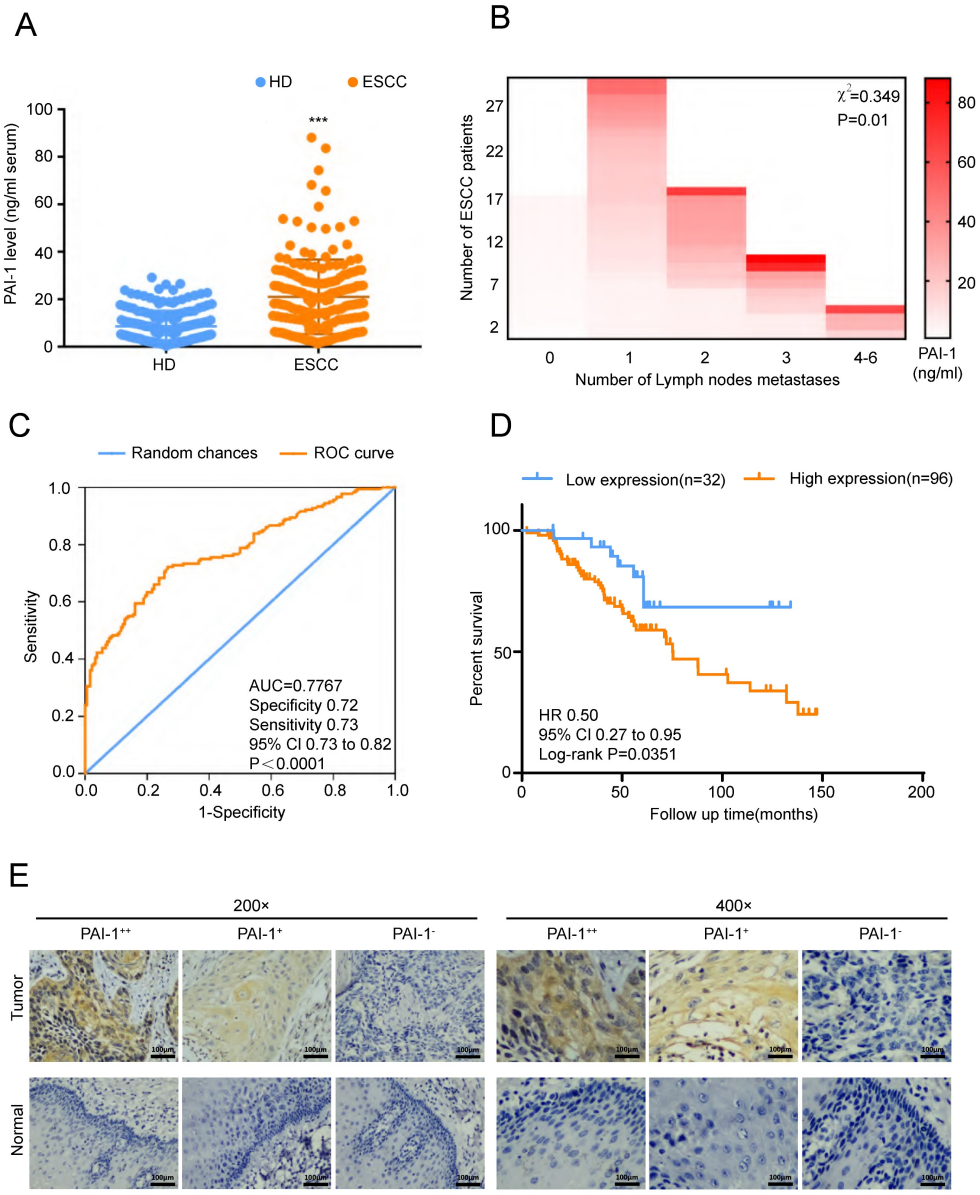
** PAI-1 expression in patients with ESCC and the clinicopathological characteristics of ESCC. A.** The level of PAI-1 in serum of ESCC patients was higher than in that of healthy donors (HD group = 8.61 ± 0.4909, n=180; ESCC group = 21.07 ± 1.17, n=180). **B.** Serum PAI-1 level was significantly higher in ESCC patients with more lymph node metastasis. **C.** ROC curve of PAI-1. Based on the area under the ROC curve, Youden Index cutoff values that maximized the sum of sensitivity and specificity were determined. **D.** Kaplan-Meier analysis of the survival rates of patients with ESCC in relation to serum PAI-1 protein expression. **E.** Representative images of PAI-1 staining according to the expression level in ESCC specimens. ***P<0.001.

In order to verify the role of PAI-1 in ESCC, we firstly detected serum PAI-1 level from 180 ESCC patients and 180 HD (health donors). We found that PAI-1 concentration in serum of ESCC was significantly higher than in that of HD (Fig. 2A). Moreover, serum PAI-1 level was markedly correlated with lymph node metastasis (Fig. 2B). ROC curve of PAI-1 was drawn based on the area under the ROC curve, and Youden Index cutoff values that maximized the sum of sensitivity and specificity were determined (Fig. 2C). Kaplan-Meier analysis of the survival rates showed that the patients with high levels of serum PAI-1 had a worse prognosis than those with low levels of PAI-1 (Fig. 2D). In addition, ESCC tissue microarray from another cohort was detected using mAb-15C2. We found expression of PAI-1 were positively associated with lymph node metastasis and negatively associated with differentiation (Fig. 2E, Tables 1-2). Thus, PAI-1 overexpression in ESCC tissues and serum samples correlates with ESCC aggressiveness.

### PAI-1 promotes migration and invasion of esophageal cancer cells

**Figure 3:**
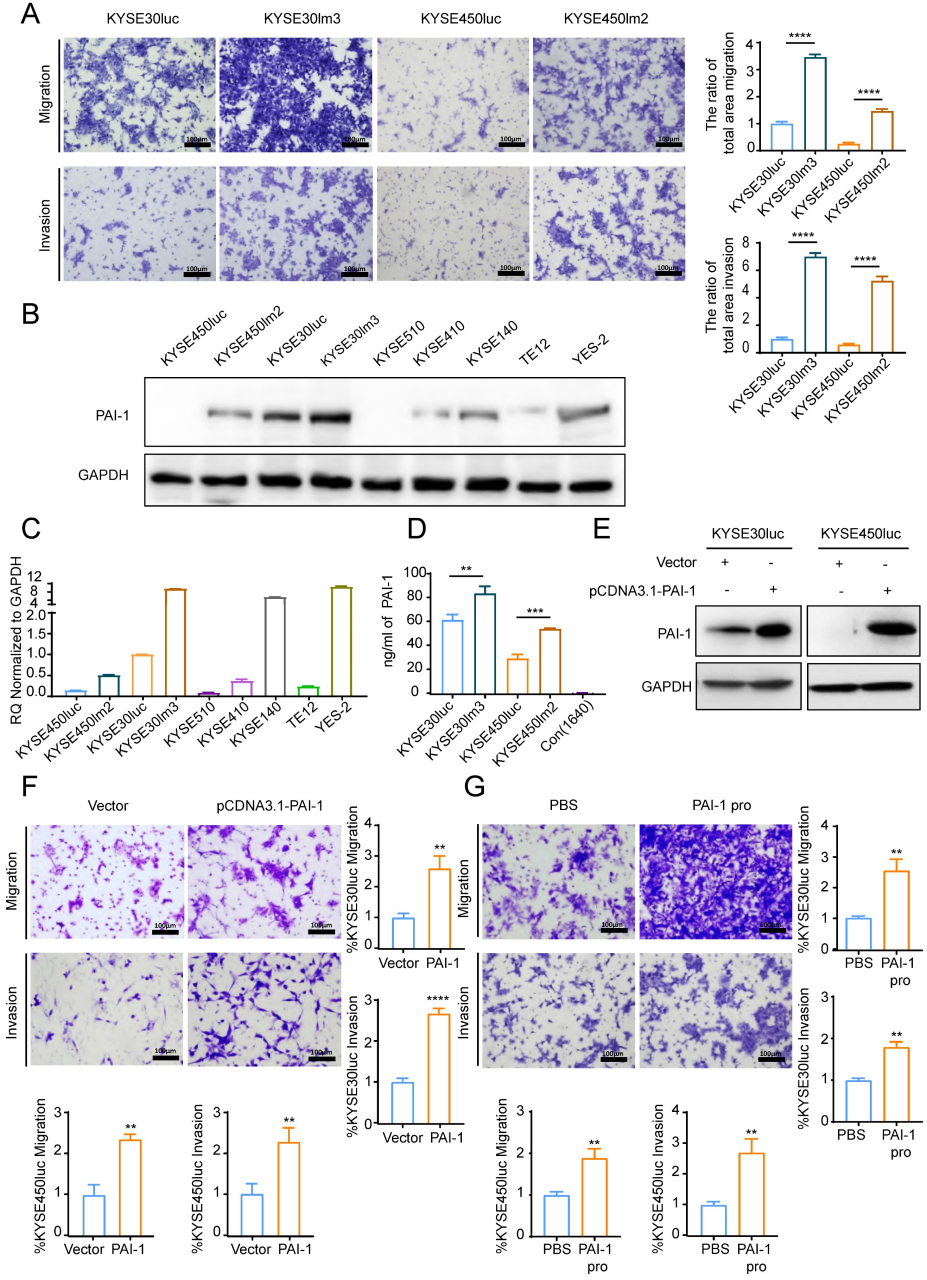
** PAI-1 promotes ESCC cell migration and invasion. A.** Transwell chamber assays and Matrigel invasion assays for the high metastasis cell lines KYSE30lm3, KYSE450lm2 and the low metastasis cell lines KYSE30luc, KYSE450luc. **B-C.** PAI-1 protein (B) and mRNA (C) level in ESCC cell lines. **D.** Levels of exocrine PAI-1 protein of indicated cells were detected by ELISA. Cells were cultured in medium lacking FBS (starvation medium) for 24h and medium was collected. **E.** PAI-1 protein level in KYSE30luc and KYSE450luc cells with PAI-1 overexpression. GAPDH was used as a loading control. **F.** Transwell chamber assays and Matrigel invasion assays for KYSE30luc and KYSE450luc cells overexpressing PAI-1. **G.** Effects of recombinant human PAI-1 (100 ng/mL) promotes on the migration and invasion of KYSE30luc and KYSE450luc cells. *P<0.05, **P<0.01, ****P<0.0001.

**Figure 4:**
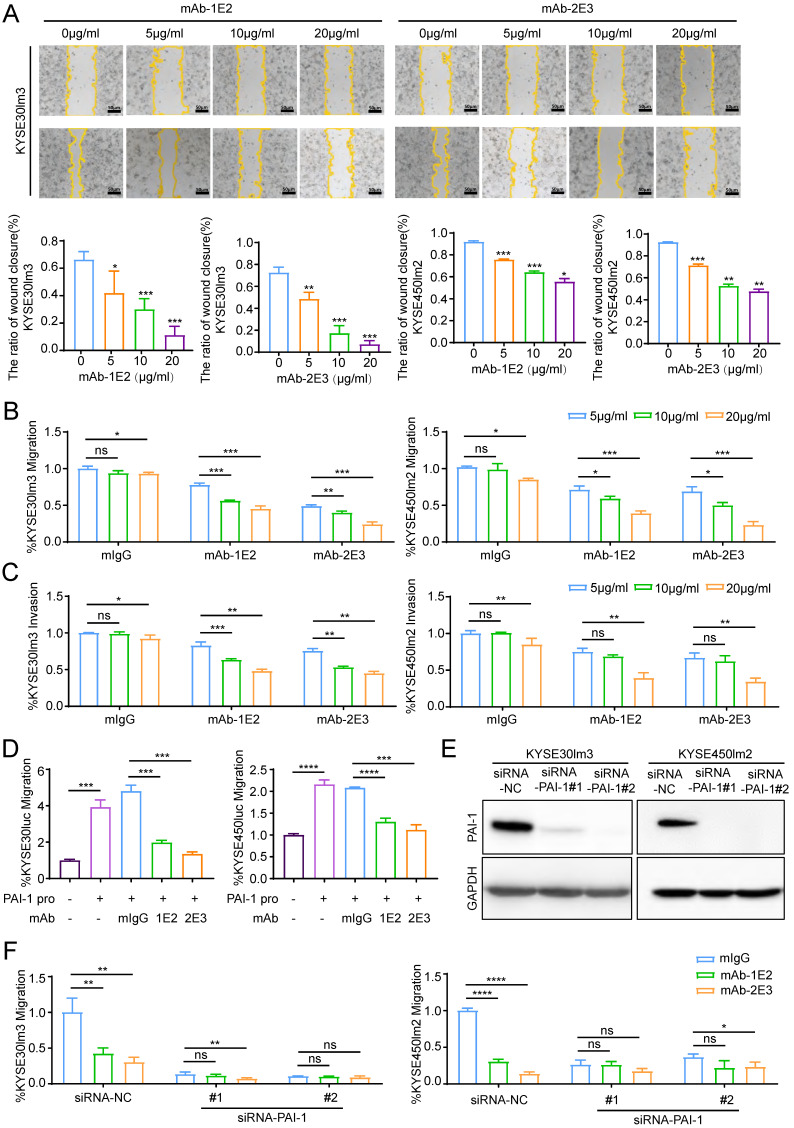
** PAI-1 mAbs inhibit the migration and invasion of KYSE30lm3 and KYSE450lm2 in a dose-dependent manner. A.** mAb-1E2 and 2E3 inhibited the wound healing of KYSE30lm3 and KYSE450lm2 in a dose-dependent manner (5 μg/mL-20 μg/mL). Bars, 50 μm. **B-C.** Inhibitory effect of mAb-1E2 and 2E3 on KYSE30lm3 and KYSE450lm2 cells by Transwell chamber assays (B) and Matrigel invasion assays (C). **D.** Inhibition of exogenous recombinant human His-PAI-1-induced cell metastasis ability with anti-PAI-1 mAbs. KYSE30luc and KYSE450luc cells were cultured for 24h in serum-free medium containing PAI-1 protein (100 ng/mL) and mAb-1E2, mAb-2E3 (20 μg/mL) or control non-immune IgG (20 μg/mL). **E.** Knockdown of PAI-1 in KYSE30lm3 and KYSE450lm2 cells. **F.** Inhibitory effects of mAb-1E2 and 2E3 on migration was abolished in PAI-1 knockdown cell lines. *P<0.05, **P<0.01, ***P<0.001, ****P<0.0001, ns ≥0.05.

To examine the impact of PAI-1 on the malignant phenotypes of ESCC cells, we utilized KYSE30lm3 and KYSE450lm2 (high metastatic ability) plus the corresponding KYSE30luc and KYSE450luc (low metastatic ability) cells. As revealed in Fig. 3A, KYSE30lm3 and KYSE450lm2 showed an enhanced migration and invasion ability than their parental cell lines. To explore the relationship between PAI-1 expression level and metastatic ability of cells, abundance and transcript level of PAI-1 in these two pairs of cell lines and other ESCC cell lines were detected. It was found that higher levels PAI-1 at both protein and transcript in KYSE30lm3 and KYSE450lm2 compared with low metastatic ability cells (Fig. 3B-C). ELISA analysis of PAI-1 in the conditioned medium (CM) from the same number of cells were showed that the exocrine PAI-1 were also higher in the high metastatic ability cells (Fig. 3D). We further overexpressed PAI-1 or added exogenous recombinant active PAI-1 protein in KYSE30luc and KYSE450luc and found that cell migration and invasion were promoted (Fig. 3E-G). Conversely, knockdown of PAI-1 inhibited cell migration and invasion (Fig. 4E-F). In addition, cell proliferation was determined by CCK8. Overexpression of PAI-1 significantly promoted cell proliferation, but the addition of exogenous protein did not (Supplemental Fig. S4A-B). Taken together, these data indicated that PAI-1 is essential for ESCC migration and invasion.

### PAI-1 mAbs inhibit the migration and invasion of PAI-1^hi^ cells

Functional assays were performed to evaluate the effects of anti-PAI-1 mAbs on the malignant phenotypes of ESCC cells. Results of wound healing assay showed that both mAb-1E2 and 2E3 inhibited motility of KYSE30lm3 and KYSE450lm2 cells in a dose-dependent fashion (Fig. 4A). These two mAbs also dose-dependently inhibited the migration and invasion of KYSE30lm3 and KYSE450lm2 cells in the Transwell chamber assays (Fig. 4B-C). mAb-1E2 and mAb-2E3 inhibited the motility of KYSE30luc cell in the wound healing assay and migration assay. However, they had no effects on that of PAI-1 deficient cell line KYSE450luc (Supplement Fig. S5A-B). Furthermore, mAb-1E2 and mAb-2E3 prevented exogenous active PAI-1-induced cells migration (Fig. 4D). The CM from EC0706 cells overexpressing PAI-1 could promote migration of KYSE30luc and KYSE450luc cells, which was abrogated by immuno-depletion of secreted PAI-1 with mAb-1E2 and mAb-2E3 (Supplement Fig. S5D-G). Through knockdown of PAI-1 in KYSE30lm3 and KYSE450lm2 cells (Fig. 4E), the inhibitory effects of mAb-1E2 and mAb-2E3 on cell migration were abrogated (Fig. 4F). In the proliferation assay, these two mAbs exhibited strong inhibit on KYSE30lm3 cells and weak inhibition on KYSE450lm2 cells (Supplement Fig. S6A). With mAb-2E3, we found that colony formation ability of ESCC cells were decreased (Supplement Fig. S6B).

### Anti-PAI-1 antibody inhibits ESCC metastasis to the lung and tumor growth in mouse models

**Figure 5:**
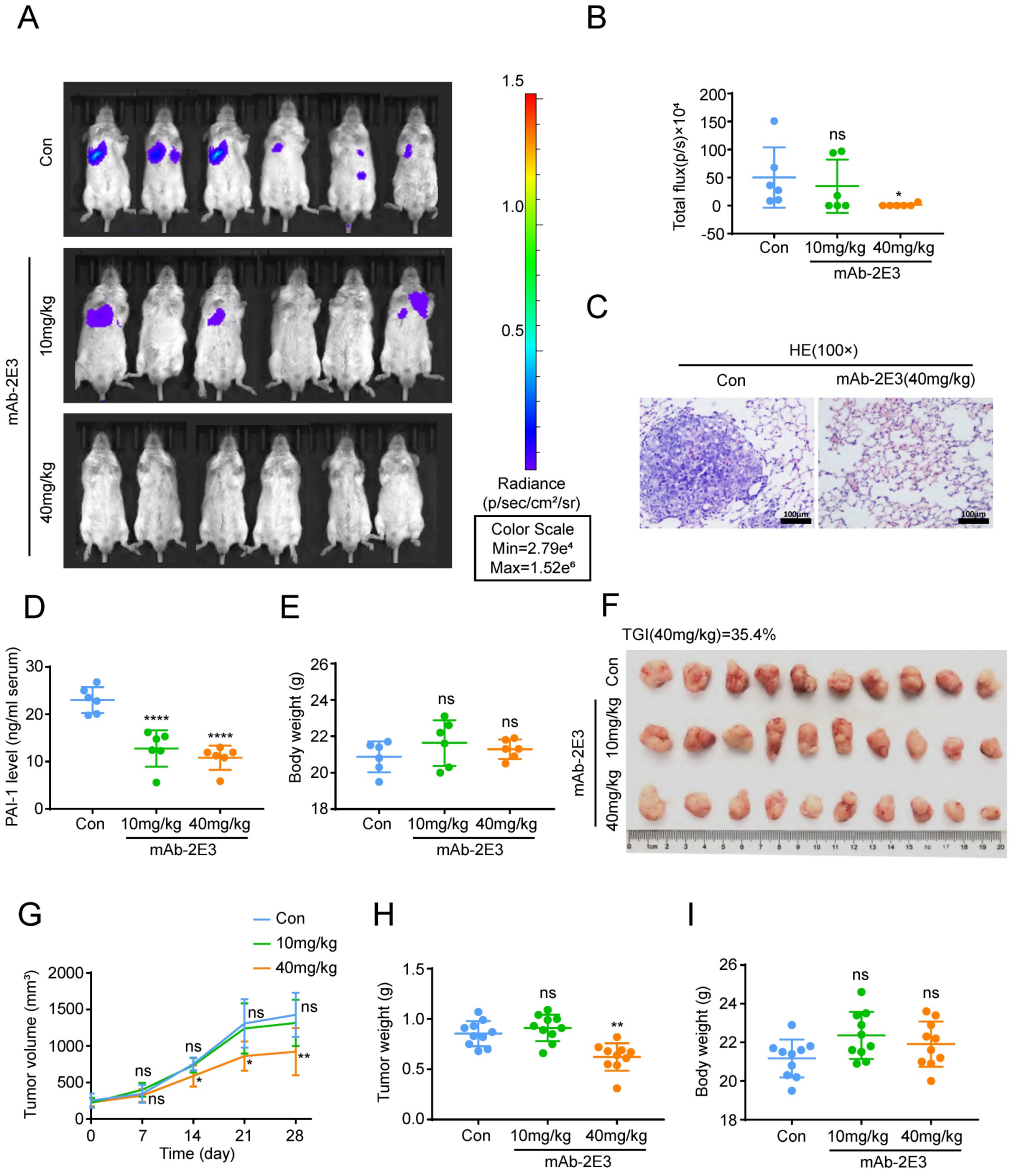
** Inhibitory effects of mAb-2E3 on the lung metastasis and tumor growth in mouse model. A-E.** Effect of mAb-2E3 on lung metastasis in SCID/Beige mice. (A) The mice were intraperitoneally injected with antibody (10 mg/kg, 40 mg/kg) or 40mg/kg control non-immune IgG every three days for ten times at 1 day after intravenous injection of 2×10^6^ KYSE30lm3 cells, and metastasis was monitored by IVIS imaging system for 4 weeks. (B) Effect of mAb-2E3 on lung metastases in the mice by IVIS. Removing extreme values (maximum and minima) for each group were shown and compared. (C) HE staining of mice lung tissues. Bars, 100μm. (D) Detection of PAI-1 level in plasma of lung metastasis mice model by ELISA. (E) Comparison of the mice weight (*P*=0.803). **F-I.** Effect of mAb-2E3 on KYSE30lm3 tumor growth in SCID/Beige mice. (F) 1×10^6^ KYSE30lm3 cells were inoculated into the flank of SCID/Beige mice. The tumors were harvest after a month of treatment with mAb or control. (G) The tumor volume (mm^3^) in the mAb group decreased significantly compared with the control. (H) Comparison of the tumor weight. (I) There was no difference in the weight of the mice. *P<0.05, **P<0.01, ****P<0.0001, ns ≥0.05.

Above results clarified that mAb-1E2 and mAb-2E3 could inhibit the malignant phenotypes of ESCC with high PAI-1 expression and the effects of mAb-2E3 were potent than those of mAb-1E2 in the *in vitro* assays. To determine the efficacy of mAb-2E3 in suppression of metastasis *in vivo*, we injected KYSE30lm3 cells through tail vein in SCID/Beige mice. The results showed that the fluorescence intensity of metastatic foci in the lungs of the 40 mg/kg mAb-2E3-treated mice was significantly lower than that in the control group (Fig. 5A-B), which was confirmed by diminished tumor burden through HE staining of lung tissues (Fig. 5C). While the fluorescence intensity of metastatic foci in the lungs of the 10 mg/kg mAb-2E3 treated mice was not statistically different from that in the control group. The serum of mice was also collected and PAI-1 was measured in serum samples by two-antibody sandwich ELISA assay. As anticipated, serum levels of PAI-1 were significantly lower in mAb-2E3-treated group than those in control group (Fig. 5D). Meanwhile, no obvious difference in the body weights of mice was found (Fig. 5E). Alternatively, a tumor-bearing model was established with KYSE30lm3 cells and the treatment started one week after tumor inoculation. The tumor volume and weight were measured at the end of treatment, and significantly lower values were obtained in the 40 mg/kg mAb-2E3-treated group, with a 35.4% of tumor growth inhibition rate (Fig. 5F, 5H). However, no decline was observed in the 10mg/kg mAb-2E3-treated group. The results showed that the tumor growth in the 40 mg/kg mAb-2E3-treated group was slower than that in the control group (Fig. 5G). Similar to the results of metastasis model, there was no significant difference in body weight was detected between the two groups (Fig. 5I), suggesting that the antibody treatment was well tolerated.

### PAI-1 mAb inhibited metastasis in an LRP1-dependent manner

**Figure 6:**
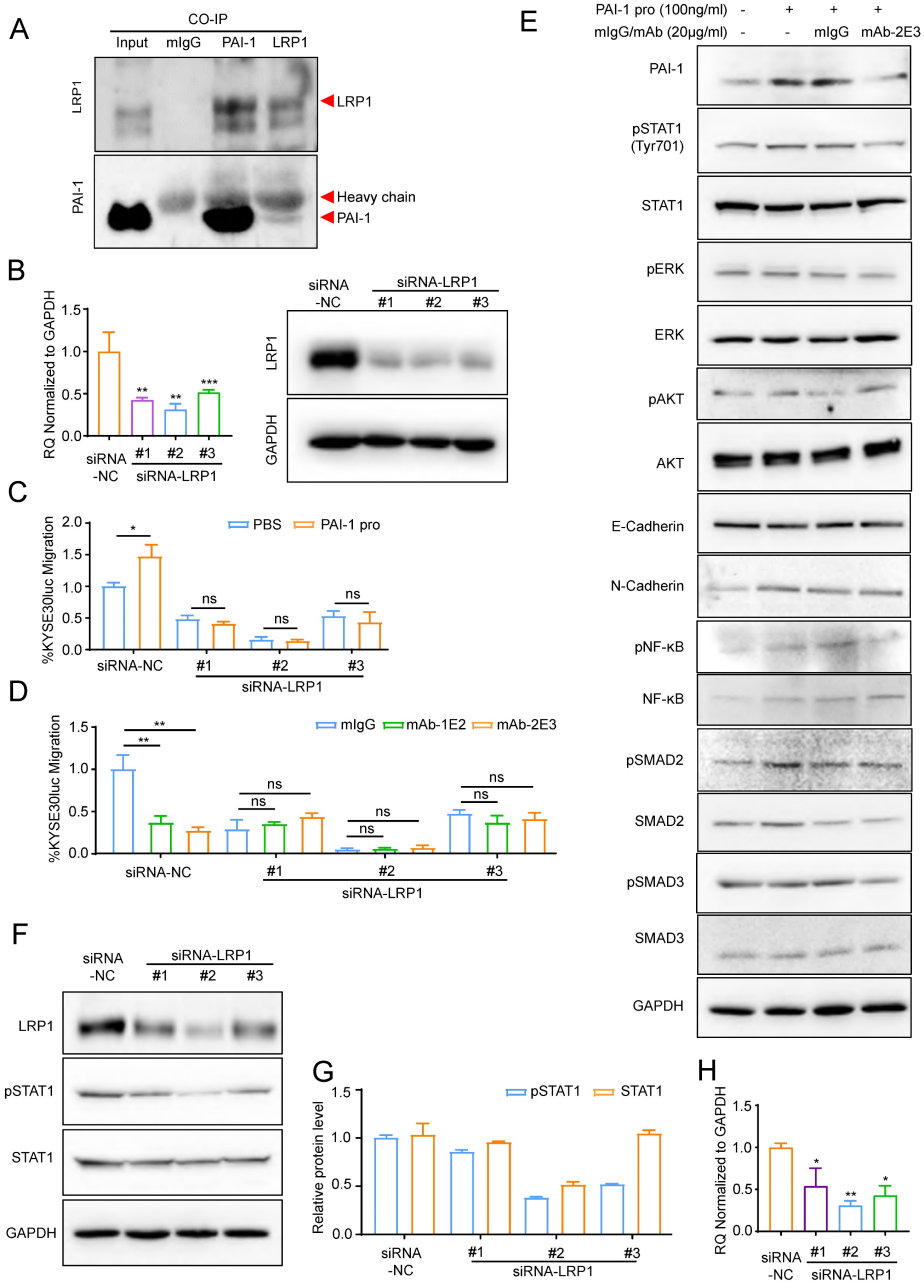
** PAI-1 mAb inhibited migration in an LRP1-dependent manner. A.** Interaction between PAI-1 and LRP1 detected by co-immunoprecipitation. **B.** qRT-PCR (left) and Western blot (right) analysis of LRP1 knockdown in KYSE30lm3 cells. **C.** Knockdown of LRP1 counteracted PAI-1 protein-promoted migration. **D.** Knockdown of LRP1abolished mAb-1E2 and 2E3-inhibited migration. **E.** mAb-2E3 inhibited the phosphorylation level of STAT1 and failed to inhibit the level of metastasis-related target proteins which were detected in cells under the treatment of antibody. **F.** Western blot of LRP1, pSTAT1 and total STAT1 in KYSE30lm3 cells transfected with siRNAs against LRP1. GAPDH was used as a loading control. **G.** Grayscale analysis of pSTAT1 and total STAT1 levels in KYSE30lm3 cells transfected with siRNAs against LRP1. **H.** qRT-PCR of STAT1 transcripts in KYSE30lm3 cells transfected with siRNAs against LRP1. *P<0.05, **P<0.01, ***P<0.001, ns ≥0.05.

It has been reported that anti-PAI-1 antibodies can neutralize PAI-1 through blocking the effect of its binding proteins 34-35. The expression level of PAI-1 ligands was measured and we found that among the four tested ligands, including VTN, LRP1, PLAU (uPA) and PLAT (tPA), LRP1 was the only one whose expression in the KYSE30lm3 and KYSE450lm2 cells was higher than that in KYSE30luc and KYSE450luc cells (Supplement S7A), which was the same as the trend of PAI-1 (Fig. 3B). Immunoprecipitation assay confirmed binding of PAI-1 with LRP1 (Fig. 6A). After silencing of LRP1 with siRNAs (Fig. 6B), both exogenous PAI-1 protein-promoted migration and anti-PAI-1-inhibited migration were abrogated (Fig. 6C-D), suggesting that the effects of PAI-1 and anti-PAI-1 mAb are LRP1-dependent. Facilitated by LRP1, PAI-1 could activate downstream signaling to boost invasiveness 36. We detected the level of metastasis-related target proteins in cells under the treatment of antibody. The results showed that the mAb-2E3 was able to inhibit the phosphorylation of STAT1 activated by PAI-1 (Fig. 6E). However, the phosphorylation levels of ERK and AKT associated with metastasis were not stimulated by active PAI-1 and inhibited by mAb-2E3 (Fig. 6E). We further examined the changes of total STAT1 and p-STAT1 after LRP1 knockdown. Results showed that knockdown of LRP1 could slightly down-regulate the total protein of STAT1, and the more complete the knock-down of LRP1, the greater down-regulation of STAT1 phosphorylation levels (Fig. 6F-H). These results were consistent with the role of PAI-1-LRP1 complex in eliciting JAK/STAT signaling 37.

### MAb-2E3 blocks the binding between LRP1-Cluster II-Fc and PAI-1

**Figure 7:**
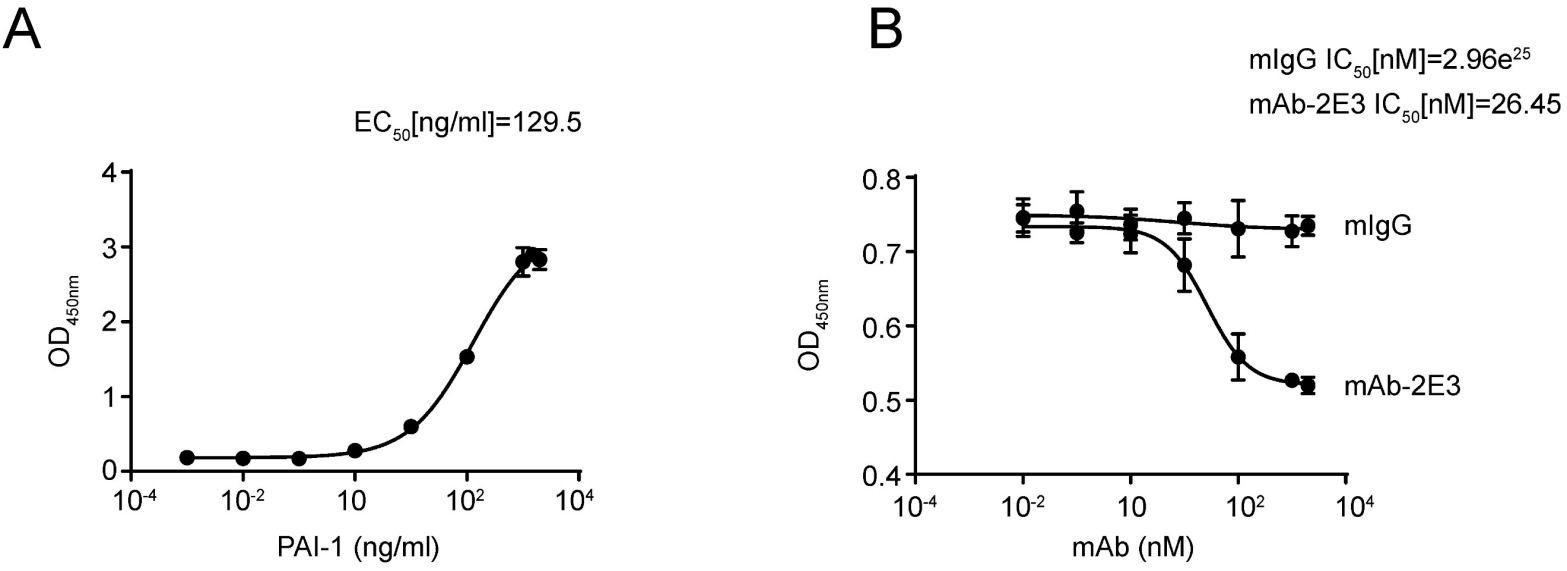
** MAb-2E3 blocking the binding of LRP1-Cluster II-Fc and PAI-1. A.** The binding activity of PAI-1 and LRP1-Cluster II-Fc protein was determined and EC50 value was calculated. **B.** mAb-2E3 was able to block the binding between LRP1-Cluster II-Fc and PAI-1. mIgG was used as the control. IC50 values were calculated.

A large number of reports indicate that anti-PAI-1 antibodies play their roles by blocking the binding between PAI-1 and its interacting molecules 38,39. Since mAb-2E3 functions through an LRP1-dependent manner, we used the LRP1-Cluster II-Fc protein, which has been shown to bind to active PAI-1, to test whether mAb-2E3 could block the binding between them. The LRP1-Cluster II-Fc protein was coated in the microtiter plate, and the binding activity of PAI-1 was detected, with the EC50 value of 129.5 ng/mL (Fig. 7A). Importantly, pre-incubation with mAb-2E3 was able to block the binding between LRP1-Cluster II-Fc and PAI-1, with the IC50 value of mAb-2E3 was 26.45 nM (Fig. 7B), while the IC50 value of mIgG was 2.96e^25^ nM.

In addition, most PAI-1 neutralizing antibodies and inhibitors have been reported to inhibit tumor metastasis by blocking the binding of uPA to PAI-1 38,40. In order to test whether the mAb-2E3 blocking the binding of uPA to PAI-1, an experiment was designed according to the role of PAI-1 in the plasminogen system. This assay adopted the classical PAI-1 activity detection method, that is, Glu-plasminogen is converted into plasmin under the activation of plasmin and zymolytic chromogenic substrate S-2251. uPA bound by active PAI-1 lost its plasminogen-activating properties. When the antibody has the effect of blocking the binding between uPA and PAI-1, S-2251 can still generate chromogen in this system 41. However, in our experimental results, the negative control mIgG and mAb-2E3 showed no difference in the coloration of S-2251, indicating that mAb-2E3 failed to block the binding between PAI-1 and uPA (Supplement S7B).

## Discussion

It has been reported that PAI-1 is highly expressed in the tissues of patients with ESCC and predicts poor prognosis 30. PAI-1 has been found to be associated with tumor metastasis in a variety of tumors 21, but the contribution of PAI-1 to metastasis of ESCC is unknown. Moreover, the correlation between serum PAI-1 levels in ESCC patients and metastasis remains unclear. In this study, we generated anti-PAI-1 monoclonal antibodies and developed a sandwich ELISA detection system, and for the first time, detected PAI-1 level in the serum of patients with ESCC. In addition, expression of PAI-1 was examined in ESCC tissue sections. The results of these two assays showed that PAI-1 in tumor tissue and serum of patients with ESCC was positively associated with metastasis.

Previous studies showed that anti-PAI-1 antibodies were initially developed as antithrombotic drugs based on the roles of PAI-1 in the plasminogen system. These antibodies prevented PAI-1 from binding to uPA or tPA by neutralizing effect, and used in thrombolytic therapy 34,35. Since the discovery of the PAI-1 in promoting tumor growth and metastasis, small molecule inhibitors of PAI-1 had been developed, including PAI-039, Tiplaxtinin, TM 5275, XR5967, and AS3288802 42,43,44. These inhibitors were demonstrated anti-tumor activity in mouse tumors. For example, Tiplaxtinin not only synergized with anti-PD-L1 in prolonging survival in a mouse model of melanoma 45, but also played synergistic effects with cisplatin in inhibiting ESCC *in vitro* and *in vivo* 46-47. XR5967 inhibited human fibrosarcoma cell migration, invasion and angiogenesis *in vitro* 43. However, none of these inhibitors have been tested in clinical trials for cancer therapy. In terms of anti-PAI-1 antibodies, monoclonal antibody MAI-12 could inhibit the migration and invasion of melanoma cell lines, but the concentration of the antibody used in the *in vitro* experiments was as high as 100 μg/mL, and it has not been evaluated in the animal experiments 46. Regarding the study by Roca et al, hyperthermia inhibited tumor angiogenesis when it combined with neutralizing antibodies MA-33HIF7 or MA-31C9 in a mouse model 47. In this study, we reported the preparation and characterization of the antibodies mAb-1E2 and mAb-2E3 with high specificity and affinity against PAI-1. Both antibodies were able to inhibit the migration and invasion of ESCC with high expression of PAI-1 *in vitro*, and mAb-2E3 showed good therapeutic effects in mouse lung metastasis and subcutaneous tumor-bearing model.

LRP1, Low density lipoprotein receptor-related protein 1, which is a large endocytic receptor capable of interacting with more than 35 ligands, mediating intracellular signaling, lipid homeostasis and apoptotic cell clearance 48. In tumor cells, LRP1, as the endocytic receptor of MMP2 and MMP9, can make malignant cells in an adherent state by activating ERK and inhibiting the JNK signaling pathway, which is conducive to invasion 49,50,51. Furthermore, the interaction of PAI-1 and LRP1 can directly lead to intracellular signaling transduction and cell migration. Studies have shown that tumor fibroblast-derived PAI-1 in esophageal cancer can induce AKT and ERK1/2 signaling pathways through LRP1 and promote cell metastasis 23,52. In addition, the binding of PAI-1 and LRP1 can stimulate vitronectin expression in tumor cells, which further regulates cell motility 18. Endometrial cancer studies by Kost also showed that ligands which are internalized with LRP1 can lead to downregulation of smad4 at both transcriptional and post-translational levels, which in turn attenuates TGF-β-related transcriptional programs and causes metastasis of endometrial cancer 53. The interaction between LRP1 and PAI-1 can promote the endocytosis of PD-L1 on the surface of melanoma cell membrane and enhance immune escape. Therefore, blocking the binding of LRP1 and PAI-1 can indirectly inhibit tumor metastasis 45. In addition, it was reported that anti-PAI-1 antibodies and inhibitors which can neutralize PAI-1 by binding to RCL competitively with PA 38,40. There are also RNA aptamers targeting PAI-1 which block its interaction with vitronectin, and play a role in anti-metastasis in an *in vitro* breast cancer cell model 54,55. However, currently there were no reports regarding anti-PAI-1 antibody or inhibitors play the anti-metastasis role through LRP1-dependent manner and block the binding of PAI-1 and LRP1. Herein, for the first time, we showed that anti-PAI-1 mAbs inhibit ESCC motility in an LRP1-dependent manner, and mAb-2E3 blocks the binding of PAI-1 and LRP1-Cluster II domain. Excess exocytosis of PAI-1 results in the direct binding of uPA/uPAR complexes to PAI-1 and endocytosis by LRP1. Intracellular degradation of the complex and recycling of uPAR to the cell surface to bind to vitronectin could accelerate cell motility 56,57. Blocking the LRP1-dependent pro-motility effect of PAI-1 can simultaneously inhibit the degradation of uPA/uPAR complex and uPAR recycling, thereby inhibiting cell motility. In addition, according to previous reports, PAI-1 neutralizing antibodies and small-molecule inhibitors have been developed that mainly act on the RCL domain of PAI-1, that is, play a neutralizing effect by blocking the binding of uPA to PAI-1 58. Most of these drugs targeting the binding sites of uPA and PAI-1 are used to inhibit tumor cell growth and promote apoptosis, and there are few reports on tumor metastasis, especially *in vivo* experiments 58,59. Moreover, the effective concentration of these inhibitors *in vitro* is high, and it is impractical to apply them *in vivo* alone 47. The LRP1-dependent anti-PAI-1 antibody we developed in this study has the effect of inhibiting metastasis without masking the binding sites of PAI-1 and uPA, which satisfies the conditions for combined therapy with these inhibitors. That is, blocking the binding of PAI-1 to LRP1 and uPA at the same time through combined therapy can inhibit tumor growth and metastasis and signal pathway activation from multiple aspects, thereby improving the therapeutic effect. Furthermore, the recent studies have shown that PAI-1 has a role in recruiting and polarizing macrophages in the tumor microenvironment, suggesting that combining anti-PAI-1 antibodies with combination therapy, including tumor immunotherapy, may achieve better treatment effect 45,49,60.

In conclusion, our findings not only further verified the potential of PAI-1 as a therapeutic target of metastatic ESCC, but also characterized a functional antibody that inhibits metastasis of ESCC *in vitro* and *in vivo*. We further showed that mAb-2E3 function through a LRP1-dependent manner and inhibit the STAT1 phosphorylation, and it has the effect of blocking the binding between LRP1-Cluster II domain and PAI-1. Overall, our findings can provide new therapeutic opportunities for PAI-1-targeted ESCC antibody therapy through further structural modification and optimization.

## Supplementary Material

Supplementary figures.

## Figures and Tables

**Table 1:** Statistical analysis of correlation between clinicopathological information and PAI-1 levels in ESCC tissue microarray

Variables	Number (n=90)	PAI-1^-^ (n=23)	PAI-1^+^ and PAI-1^±^ (n=59)	PAI-1^++^ (n=8)	χ^2^	*P*
**Gender**					0.260	1.000
Male	72	18	47	7		
Female	18	5	12	1		
**Age, years**					3.734	0.178
≤60	39	12	26	1		
>60	51	11	33	7		
**Lymph nodes metastases**					7.871	0.018*
Negative	61	20	34	7		
Positive	29	3	25	1		
**Differentiation**					10.388	0.023*
G1	33	7	23	3		
G2	47	10	34	3		
G3	10	6	2	2		
**Pathology T stage**					6.739	0.244
T1	3	0	3	0		
T2	14	3	9	2		
T3	59	17	38	4		
T4	5	0	3	2		
No detected	9	3	6	0		

**Table 2:** Statistical analysis of the correlation between clinicopathological information and PAI-1 levels in sera from ESCC patients

Variables	Number (n=128)	PAI-1^low^ (n=32)	PAI-1^high^ (n=96)	χ^2^	*P*
**Gender**				1.669	0.196
Male	109	25	84		
Female	19	7	12		
**Number of Lymph nodes metastases**					
0	65	17	48	0.349	0.01*
1	31	7	24		
2	18	6	12		
3	10	2	8		
4-6	4	0	4		
**Pathology T stage**					
T1	4	1	3	0.683	0.073
T2	40	10	30		
T3	82	20	62		
T4	2	1	1		
