# Supplementary material for: Anti-PAI-1 Monoclonal Antibody Inhibits the Metastasis and Growth of Esophageal Squamous Cell Carcinoma
Source: J Cancer. 2023 Jan 1;14(1):114–28. doi: 10.7150/jca.77888 (PMC9809335; doi:10.7150/jca.77888)
Supplement: Supplementary file 1 — Supplementary figures. [file jcav14p0114s1.pdf]

Supplement Figure

S1 Screening of anti-PAI-1mAbs

A. The specificity of the reaction between anti-PAI-1 antibodies and GST-PAI-1 proteins detected by ELISA. GST and GST-PAI-1 prokaryotic protein (1 μg/mL) were coated in the 96-well plate at 100 μl/well. Anti-PAI-1 mAb were added to the wells at a concentration of 2 μg/mL and incubated for 2 h, followed by detection using TMB.

B. Immunoprecipitation of PAI-1 from KYSE30luc cells with mAbs.

C. Detection of eukaryotic PAI-1 protein (His-PAI-1) and prokaryotic PAI-1 protein (GST-PAI-1) with mAbs by Western blot.

D. Detection of eukaryotic PAI-1 protein (His-PAI-1) and prokaryotic PAI-1 protein (GST-PAI-1) with mAb-2E3 by Western blot.

S2 Identification of His-PAI-1 protein activity

A-B. Identification of His-PAI-1 protein activity by Z-GGR-AMC system **(A)** and S-2251 system **(B).**

S3 mAb pairing for detection of PAI-1 by sandwich ELISA

A. mAb-1E2 and mAb-2E3-HRP were respectively identified as capture and detection antibodies in double-antibody sandwiched ELISA for the detection of PAI-1**.**

B. Standard curve line of the PAI-1 detection**.**

S4 Effects of PAI-1 on cell proliferation.

A. Effect of PAI-1 overexpression on proliferation.

B. Effects of exogenous human recombinant His-PAI-1 (100 ng/mL) on proliferation

**$P < 0.01$, ns ≥ 0.05.

S5 PAI-1 mAb inhibits the migration and invasion of KYSE30lm3 and KYSE450lm2 in a dose-dependent manner

A. mAb-2E3 showed stronger ability in preventing migration compared to mAb-1E2.

B-C. Effects of mAb-1E2 and mAb-2E3 on the motility of KYSE30lm3 and KYSE30luc cells in Wound healing assay **(B)** and Transwell chamber assay **(C).**

D-F. PAI-1 in conditioned medium (CM) of EC9706 cells promotes migration, which is attenuated by immunodepletion of PAI-1. **(D)** Detection of PAI-1 protein overexpression in EC9706 cells. **(E)** Immunodepletion of PAI-1 in the CM of EC9706 cells. **(F)** Western blot analysis of PAI-1 in the CM of EC9706 cells after immunodepletion. **(G)** Immunodepletion of PAI-1 in the CM of EC9706 cells attenuated mAb-1E2 and mAb-2E3' inhibitory effects on migration.

*$P < 0.05$, **$P < 0.01$, ***$P < 0.001$, ns $P \geq 0.05$.

S6 PAI-1 mAb inhibits proliferation and colony formation of PAI-1^{hi} cells

A. Effects of PAI-1 mAbs on proliferation of KYSE30lm and KYSE450lm2 cells.

B. Effects of PAI-1 mAbs on colony formation of KYSE450lm2, KYSE30lm3 and YES-2 cells.

*$P < 0.05$, **$P < 0.01$, ***$P < 0.001$, ns $P \geq 0.05$.

S7 mAb-2E3 did not active in blocking PAI-1 site which binds to PLAU

A. Expression of PAI-1 ligands.

B. The mAb-2E3 did not affect PAI-1 activity as detected in S-2251 system, indicating that mAb-2E3 failed to block the binding of PAI-1 and uPA.

Fig.S1.

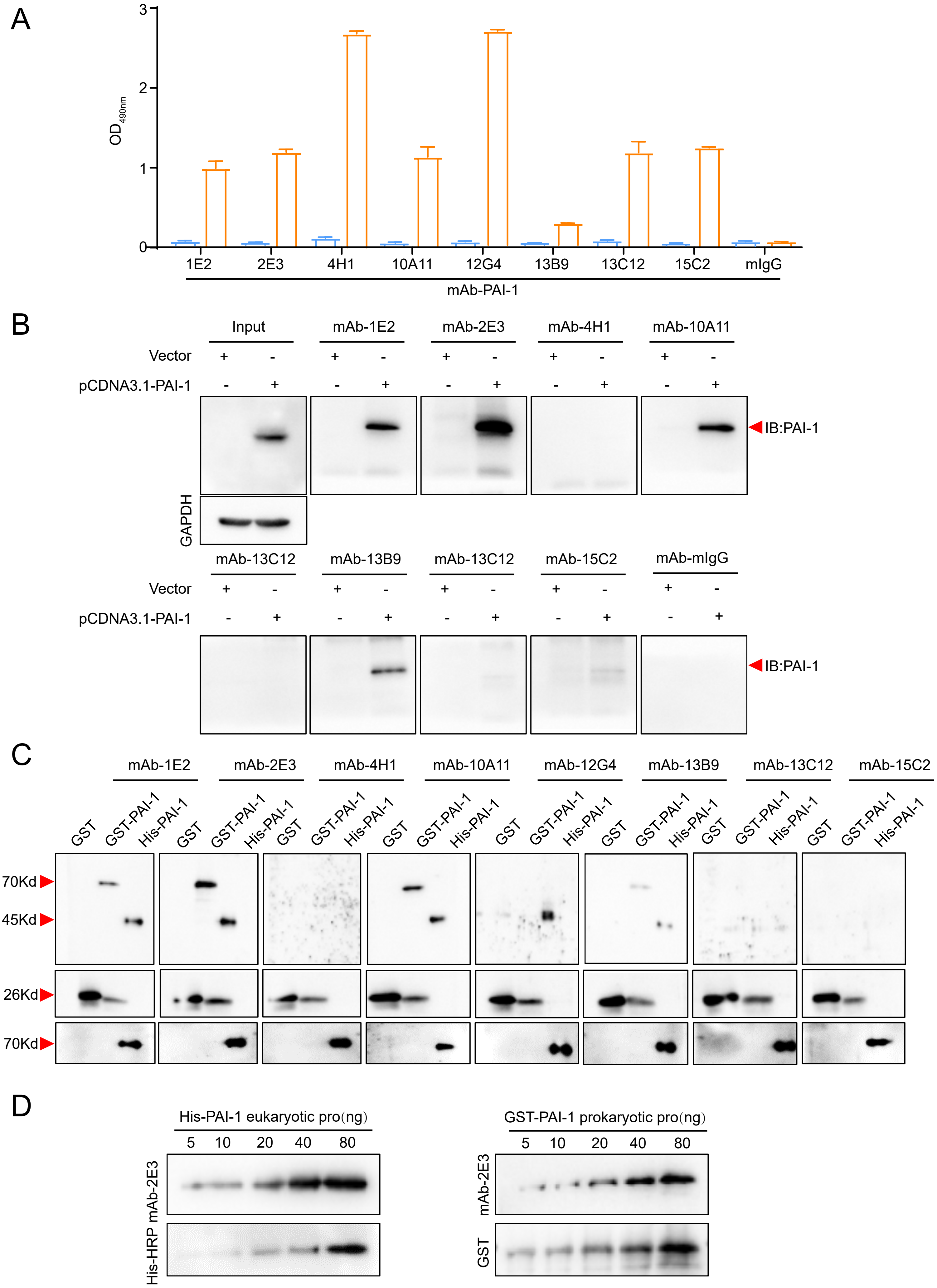
A
OD490nm
3
2
1
0
1E2
2E3
4H1
10A11
12G4
13B9
13C12
15C2
mIgG
mAb-PAI-1
B
Input
mAb-1E2
mAb-2E3
mAb-4H1
mAb-10A11
Vector
pCDNA3.1-PAI-1
IB:PAI-1
GAPDH
mAb-13C12
mAb-13B9
mAb-13C12
mAb-15C2
mAb-mIgG
Vector
pCDNA3.1-PAI-1
IB:PAI-1
C
mAb-1E2
mAb-2E3
mAb-4H1
mAb-10A11
mAb-12G4
mAb-13B9
mAb-13C12
mAb-15C2
GST
GST-PAI-1
His-PAI-1
PAI-1
70Kd
45Kd
GST
26Kd
6×His
70Kd
D
His-PAI-1 eukaryotic pro(ng)
5 10 20 40 80
mAb-2E3
His-HRP
GST-PAI-1 prokaryotic pro(ng)
5 10 20 40 80
mAb-2E3
GST

Fig.S2.

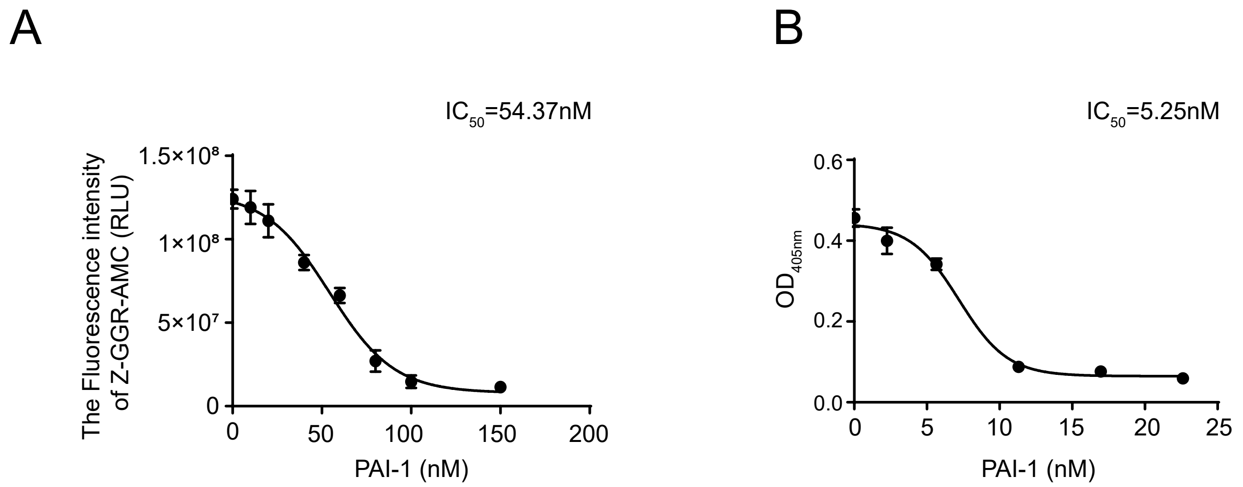
A
B
IC50=54.37nM
The Fluorescence intensity of Z-GGR-AMC (RLU)
1.5×10⁸
1×10⁸
5×10⁷
0
0
50
100
150
200
PAI-1 (nM)
IC50=5.25nM
OD405nm
0.6
0.4
0.2
0.0
0
5
10
15
20
25
PAI-1 (nM)

Fig.S3.

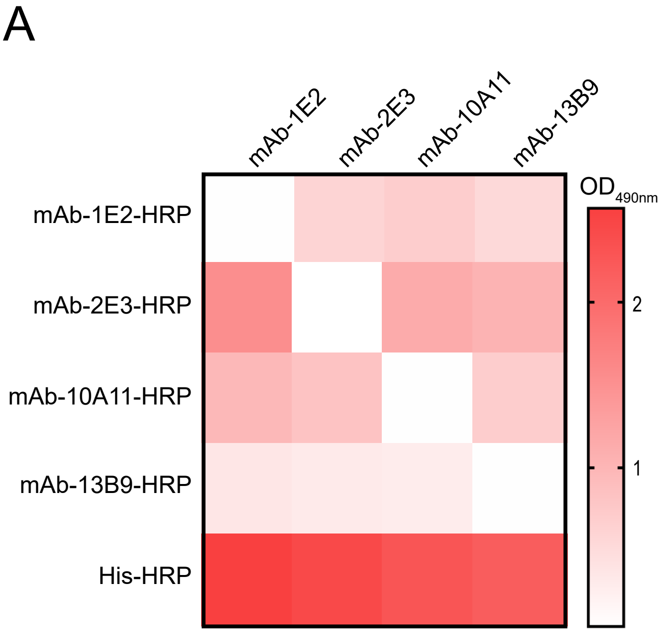
A
mAb-1E2
mAb-2E3
mAb-10A11
mAb-13B9
mAb-1E2-HRP
mAb-2E3-HRP
mAb-10A11-HRP
mAb-13B9-HRP
His-HRP
OD490nm
2
1

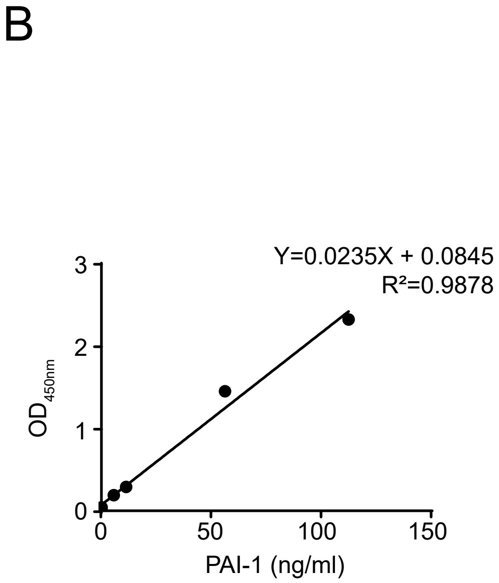
B
Y=0.0235X + 0.0845
R²=0.9878
OD450nm
3
2
1
0
0
50
100
150
PAI-1 (ng/ml)

Fig.S4.

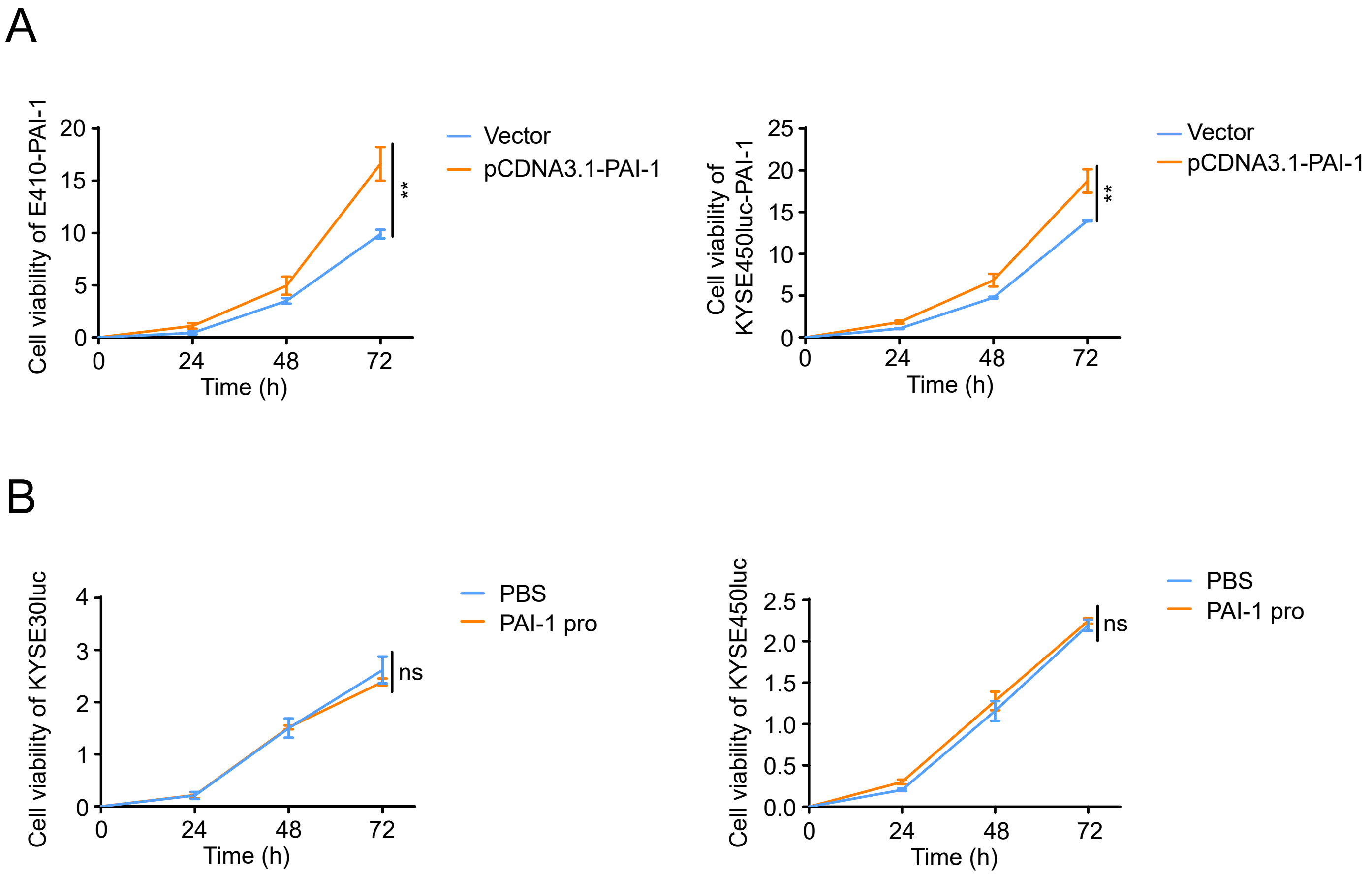
A
Cell viability of E410-PAI-1
20
15
10
5
0
0
24
48
72
Time (h)
**
Vector
pCDNA3.1-PAI-1
Cell viability of KYSE450luc-PAI-1
25
20
15
10
5
0
0
24
48
72
Time (h)
**
Vector
pCDNA3.1-PAI-1
B
Cell viability of KYSE30luc
4
3
2
1
0
0
24
48
72
Time (h)
ns
PBS
PAI-1 pro
Cell viability of KYSE450luc
2.5
2.0
1.5
1.0
0.5
0.0
0
24
48
72
Time (h)
ns
PBS
PAI-1 pro

Fig.S5.

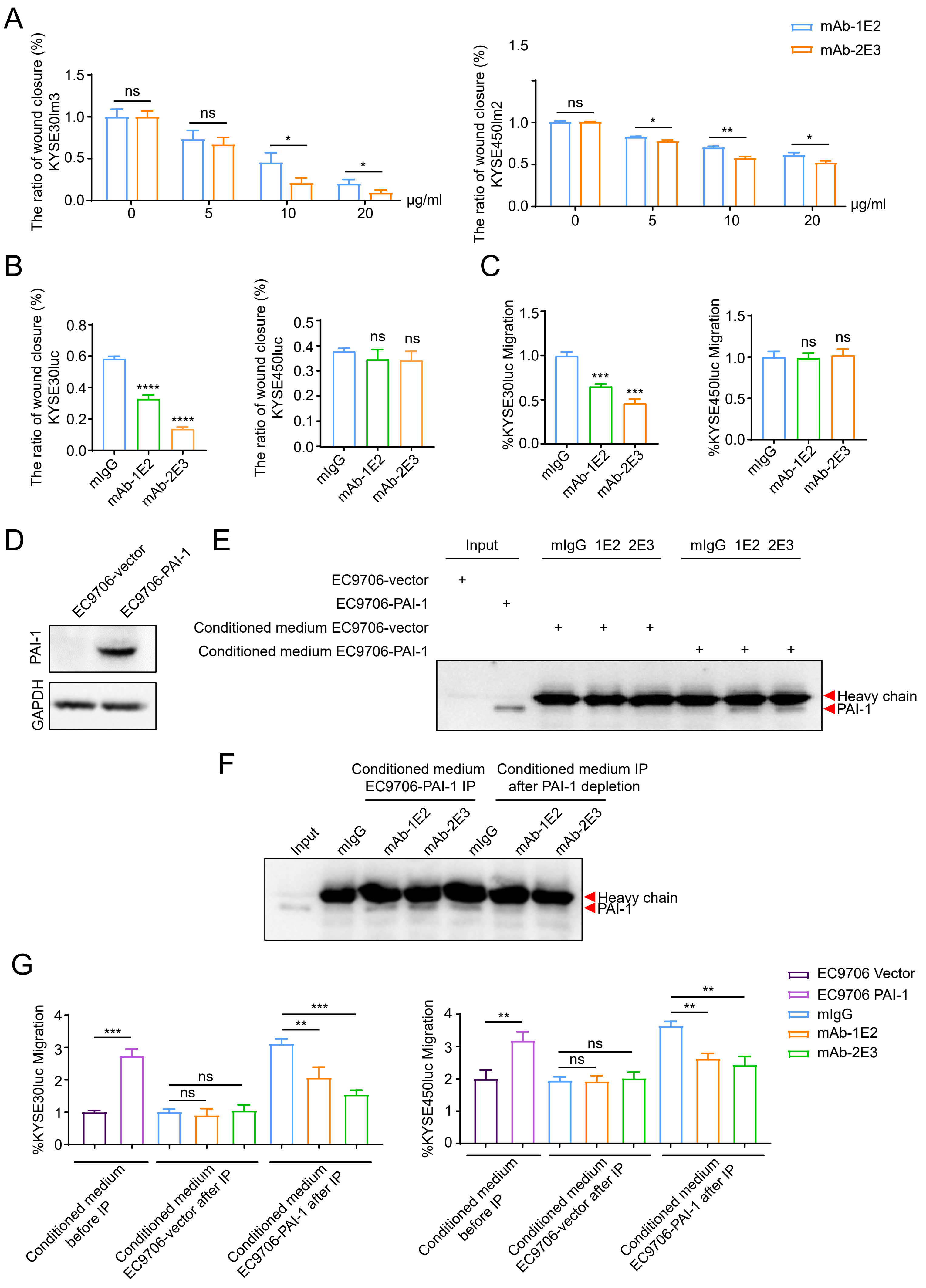

A
B
C
D
E
F
G
mAb-1E2
mAb-2E3
The ratio of wound closure (%) KYSE30lm3
The ratio of wound closure (%) KYSE450lm2
μg/ml
ns
*
**
The ratio of wound closure (%) KYSE30luc
The ratio of wound closure (%) KYSE450luc
%KYSE30luc Migration
%KYSE450luc Migration
mIgG
mAb-1E2
mAb-2E3
****
***
EC9706-vector
EC9706-PAI-1
PAI-1
GAPDH
Input
mIgG 1E2 2E3
EC9706-vector
EC9706-PAI-1
Conditioned medium EC9706-vector
Conditioned medium EC9706-PAI-1
Heavy chain
PAI-1
Conditioned medium EC9706-PAI-1 IP
Conditioned medium IP after PAI-1 depletion
EC9706 Vector
EC9706 PAI-1
Conditioned medium before IP
Conditioned medium EC9706-vector after IP
Conditioned medium EC9706-PAI-1 after IP

Fig.S6.

A

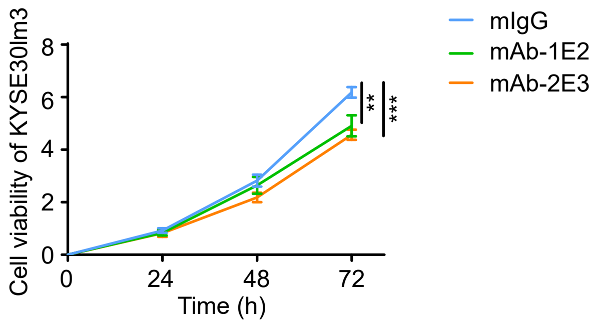

Cell viability of KYSE30lm3
8
6
4
2
0
0
24
48
72
Time (h)
**
***
mIgG
mAb-1E2
mAb-2E3

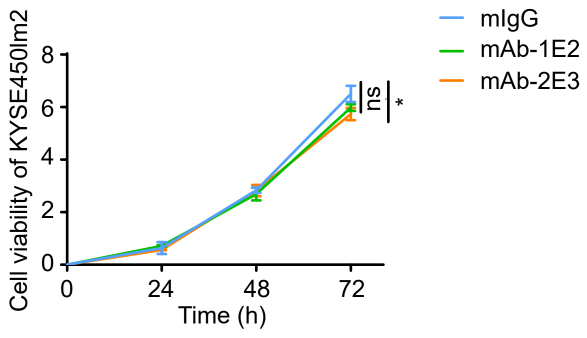

Cell viability of KYSE450lm2
8
6
4
2
0
0
24
48
72
Time (h)
ns
*
mIgG
mAb-1E2
mAb-2E3

B

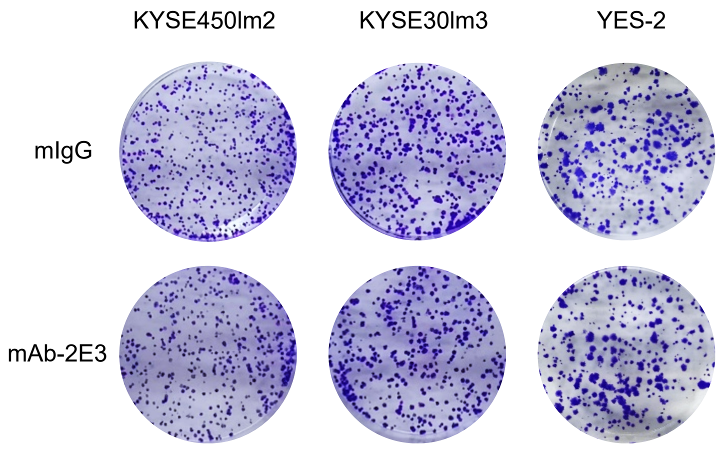

KYSE450lm2
KYSE30lm3
YES-2
mIgG
mAb-2E3

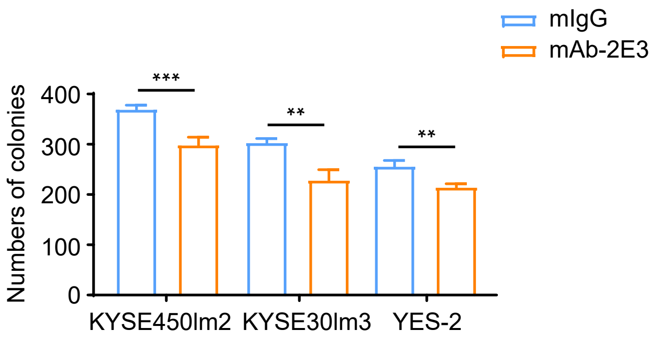

mIgG
mAb-2E3
Numbers of colonies
400
300
200
100
0
***
**
**
KYSE450lm2
KYSE30lm3
YES-2

Fig.S7.

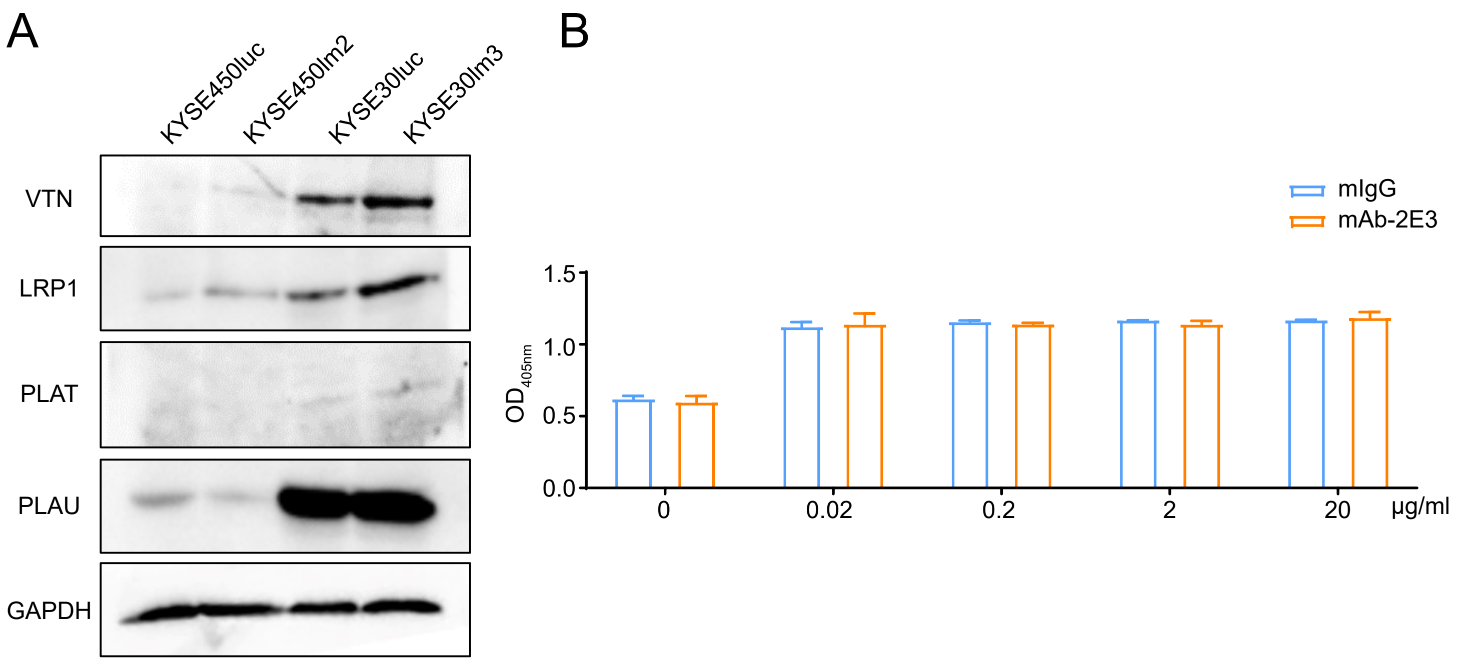
A
KYSE450luc
KYSE450lm2
KYSE30luc
KYSE30lm3
VTN
LRP1
PLAT
PLAU
GAPDH
B
mIgG
mAb-2E3
OD_{405nm}
1.5
1.0
0.5
0.0
0
0.02
0.2
2
20
μg/ml
